# Multi-Omic and Spatial Profiling Identifies an Epithelial DKK1 Associated with Microenvironmental Remodeling in Pancreatic Ductal Adenocarcinoma

**DOI:** 10.3390/cimb48020182

**Published:** 2026-02-05

**Authors:** Jiajia Xu, Kaiqiang Qian, Yanyu Ding, Jianghao Cheng, Xu Zhang, Yong Huang, Bo Liu

**Affiliations:** 1 Department of Immunology, School of Basic Medical Sciences, Center for Big Data and Population Health of IHM, Anhui Medical University, Hefei 230032, China; 2346010010@stu.ahmu.edu.cn (J.X.); yanyuding@ihm.ac.cn (Y.D.); chengjianghao@ihm.ac.cn (J.C.); zhangxu@ihm.ac.cn (X.Z.); 2 Hefei Comprehensive National Science Center, Institute of Health and Medicine, Hefei 230093, China; 3 National Engineering Laboratory of Crop Stress Resistance Breeding, School of Life Sciences, Anhui Agricultural University, Hefei 230036, China; 23710089@stu.ahau.edu.cn

**Keywords:** pancreatic ductal adenocarcinoma, DKK1, tumor microenvironment, spatial transcriptomics, single-cell RNA-seq, WGCNA, CellChat, epithelial–endothelial communication

## Abstract

**Objective:** This study aimed to identify clinically relevant regulators of pancreatic ductal adenocarcinoma (PDAC), a disease characterized by stromal remodeling and immune suppression, and to define their links to malignant progression and microenvironmental reprogramming. **Methods:** We integrated multi-cohort bulk, single-cell, and spatial transcriptomic datasets and subsequently validated bulk differential expression and network analyses with machine learning-based prioritization in an independent combined cohort (TCGA-PAAD plus GSE62452). Single-cell mapping was used to assess cell-type specificity, positioning candidates along inferCNV- and pseudotime-defined malignant continua. In Visium sections, a DKK1-associated program score quantified intratumoral spatial heterogeneity and informed our analyses of ligand–receptor communication. Bulk immune deconvolution linked gene levels to immune infiltration patterns, and functional assays were used to test the impact of DKK1 knockdown on migration, proliferation, clonogenic growth, and apoptosis in PDAC cells. **Results:** Four reproducible tumor-associated genes—DKK1, COL10A1, SULF1, and SLC24A3—were prioritized and validated externally. DKK1 was predominantly expressed by epithelial tumor cells and tracked along a malignant progression continuum. Spatially, the DKK1 program localized to epithelial-dominant regions, revealed pronounced intratumoral heterogeneity, and highlighted epithelial–endothelial and endothelial–immune signaling in high-score areas. Immune deconvolution associated higher DKK1 expression with increased myeloid infiltration and reduced cytotoxic lymphocyte signatures. Functionally, DKK1 knockdown impaired migration, proliferation, and clonogenicity while increasing apoptosis. **Conclusions:** We demonstrate that DKK1 is an epithelial-derived regulator linked to malignant progression and tumor–stroma–immune remodeling, supporting its potential as a biomarker and therapeutic target in PDAC treatment, including rational combinations with stroma-modulating strategies and immunotherapy.

## 1. Introduction

Pancreatic ductal adenocarcinoma (PDAC), the dominant histological subtype of pancreatic cancer, remains one of the deadliest solid malignancies, with a 5-year survival rate below 10% despite advances in surgery and systemic therapy [1,2]. The incidence and mortality of PDAC continue to increase globally; it is projected to become the second leading cause of cancer-related death within the next decade. This persistently poor prognosis highlights the urgent need for improving early detection and timely intervention strategies [3]. Epidemiological studies implicate lifestyle and metabolic factors in PDAC development, with cigarette smoking being a particularly well-established, modifiable risk factor; additional tobacco exposures further increase risk, and earlier initiation and longer duration are associated with higher mortality and widening disparities across populations [4,5,6,7]. Metabolic dysfunction—encompassing diabetes mellitus, obesity, insulin resistance, and impaired glucose homeostasis—also contributes to PDAC risk. Notably, new-onset diabetes is increasingly recognized as both a consequence of PDAC and a potential early clinical signal, with prospective evidence supporting its use in risk stratification [8,9,10,11].

In addition to these risk factors, PDAC progression and therapeutic refractoriness are strongly shaped by coordinated tumor–stroma–immune programs and pronounced inter- and intra-tumoral heterogeneity. Dense desmoplasia, extracellular matrix (ECM) remodeling, and immune suppression jointly constrain drug delivery and antitumor immunity, and may promote malignant-state transitions. Consequently, to pinpoint actionable regulators, a system-level framework is required that integrates bulk transcriptomic discovery with network modeling and objective prioritization, resolving cellular sources and tissue context using single-cell and spatial approaches.

Of the pathways that are repeatedly implicated in PDAC, dysregulation of Wnt/β-catenin signaling has been associated with tumor progression and microenvironmental remodeling [12]. Dickkopf-related protein 1 (DKK1) is a secreted glycoprotein that inhibits canonical Wnt signaling by binding the low-density lipoprotein receptor-related proteins 5 and 6 (LRP5/6), thereby preventing ligand–receptor complex formation. Mechanistically, the C-terminal domain of DKK1 interacts with the β-propeller region of LRP6 [13]. DKK1 is also a direct transcriptional target of the β-catenin/TCF complex, creating a negative feedback loop [14]. Consistent with this regulatory role, aberrant DKK1 expression has been linked to tumor progression across cancer types [15,16]. In PDAC, epigenomic profiling has identified a DKK1 super-enhancer that recruits AP-1 to sustain high DKK1 expression; disrupting this element suppresses tumor growth and reshapes the microenvironment [17]. Moreover, DKK1 and CKAP4 can be packaged into exosomes and detected in serum, supporting their potential as circulating biomarkers; blocking the DKK1–CKAP4 axis showed antitumor activity in preclinical models [18]. A DKK1–FOXM1 feed-forward circuit has also been reported to accelerate PDAC progression and is associated with adverse outcomes [19].

Despite these observations, key questions remain, including the predominant cellular source of DKK1 within the PDAC microenvironment, its relationship to malignant-state transitions, and its tissue-contextual communication under spatial heterogeneity. Bulk transcriptomic associations cannot determine whether DKK1 activity is primarily driven by the malignant epithelium or stromal/immune compartments, nor do they resolve intratumoral spatial patterns and their implications for intercellular crosstalk. To address these research gaps, we combined bulk transcriptomic discovery with network- and machine learning-based prioritization to nominate robust candidate regulators, and then leveraged single-cell and spatial transcriptomics to localize candidate genes at the cellular and tissue scales. Using inferCNV-guided malignant-state stratification and pseudotime reconstruction, we positioned epithelial-derived DKK1 along a continuum of malignant progression, and applied spatial program scoring and CellChat inference to delineate the communication networks associated with DKK1. Finally, we used functional perturbation assays to validate the impact of DKK1 on migration, proliferation, clonogenic growth, and apoptosis, providing multi-omic and experimental evidence for DKK1’s role as a clinically relevant biomarker and potential therapeutic target in PDAC.

## 2. Materials and Methods

### 2.1. Data Preparation

The GSE15471 cohort (GPL570, 39 normal and 39 PDAC samples) was downloaded from the Gene Expression Omnibus (GEO) and used for bulk transcriptomic discovery analyses. Candidate genes were validated in an independent integrated cohort combining TCGA-PAAD and GSE62452. Single-cell RNA-seq (scRNA-seq) datasets (GSE205013 and GSE155698, 27 primary PDAC tumors) and Visium spatial transcriptomic data (PRJNA1124001 with images/metadata from Zenodo record 13379726) were used for cell-type mapping and spatial validation.

### 2.2. Differentially Expressed Genes (DEGs) Screening

The GSE15471 expression matrix was annotated using the GPL570 platform file. Differential expression between tumor and normal samples was computed on log_2_-transformed data using limma, defining DEGs as |log_2_FC| ≥ 1 with Benjamini–Hochberg–adjusted *p* < 0.05. The results were visualized in volcano plots, and the top 50 upregulated and top 50 downregulated DEGs were further examined using Pearson correlation and displayed as heatmaps.

### 2.3. Functional Enrichment Analysis

Functional enrichment analysis of the upregulated DEGs was carried out using Gene Ontology (GO) terms and Kyoto Encyclopedia of Genes and Genomes (KEGG) pathways. The enrichment analyses were performed with DAVID Bioinformatics Resources (https://david.ncifcrf.gov, accessed on 25 January 2026), and the results were visualized using the Weishengxin online platform (https://www.bioinformatics.com.cn/, accessed on 25 January 2026).

### 2.4. Weighted Gene Co-Expression Network Analysis (WGCNA) Construction

Co-expression network analysis was performed in R using WGCNA. The soft-thresholding power was selected using pickSoftThreshold (version 1.72-5), and an adjacency matrix was converted to a topological overlap matrix (TOM) for hierarchical clustering. Modules were identified with dynamic tree cutting (minimum module size = 50), with PDAC-associated modules prioritized based on gene significance (GS) and module membership (MM).

### 2.5. Least Absolute Shrinkage and Selection Operator (LASSO) Regression Analysis

LASSO regression was performed in R using glmnet (version 4.1-10). The optimal regularization parameter (λ) was selected using five-fold cross-validation to minimize the cross-validated mean squared error, and genes with non-zero coefficients at the optimal λ were retained as candidate core biomarkers for downstream analyses.

### 2.6. Random-Forest (RF) Analysis

RF modeling was performed in R using randomForest (version 4.7-1.2), with gene expression as predictors and sample class labels as outcomes. A forest of 500 trees was fitted, and the out-of-bag error curve was used to determine an appropriate number of trees. Variable importance was extracted to rank candidate biomarkers, and the top 40 genes were visualized using bar and scatter plots.

### 2.7. Candidate Gene Validation

An integrated validation cohort combining TCGA-PAAD and GSE62452 was assembled to validate hub genes. Group-wise expression differences were assessed in R using pcutils (with supporting packages ggstatsplot, PMCMRplus, ggplot2, and cowplot), with a fixed random seed for reproducibility. Hub-gene expression was compared across groups using group_box and the data visualized as violin plots.

### 2.8. Gene Set Enrichment Analysis (GSEA) Analysis

GSEA was performed in R using clusterProfiler (version 4.12.0, org.Hs.eg.db and enrichplot), with parallel computation via future/future.apply. A genome-wide ranked list was generated by computing Spearman correlations between DKK1 and all other genes, after mapping gene symbols to Entrez IDs using bitr. Enrichment was conducted with GSEA using the GO gene set collection (c5.go.v2023.2.Hs.symbols.gmt).

### 2.9. Immune Cell Infiltration Analysis

Immune cell infiltration was estimated from bulk expression data using CIBERSORT to infer the relative fractions of 22 immune cell subsets, retaining only samples with CIBERSORT *p* < 0.05. Data preprocessing was performed using limma, reshape2, and tidyverse, and associations between gene expression and inferred immune fractions were assessed with Spearman correlation. Significant correlations (*p* < 0.05) were visualized using scatter plots with fitted trend lines and density overlays.

### 2.10. ScRNA-Seq Data Filtering and the Standard Process

A total of 27 primary PDAC tumor samples (GSE205013, n = 11; GSE155698, n = 16) comprising 154,590 cells were processed in Seurat. Cells were filtered using nFeature_RNA, nCount_RNA, percent.mt, and percent. HB; they were retained if values were 300 < nFeature_RNA < 8000, 300 < nCount_RNA < 15,000, percent.mt < 10%, and percent. HB < 1% based on violin plots and quantile distributions. Cell-cycle effects were regressed out, yielding 95,218 cells after QC; batch effects were corrected with Harmony, followed by dimensionality reduction, clustering, and cell-type annotation using SingleR (version 2.4.0).

### 2.11. InferCNV

Epithelial cells and CD8^+^ T cells were extracted from the SingleR-annotated Seurat object, and large-scale CNV profiles were inferred using inferCNV (cutoff = 0.1) with CD8^+^ T cells as the reference, based on an hg38 gene-ordering file (GENCODE v27). CNV burden was quantified per cell as the CNV score from the inferCNV output (mean of (expr-1)^2^) and summarized across epithelial clusters, which were stratified into high, intermediate, and low-malignancy groups according to cluster-level CNV burden and visualized using heatmaps and cluster-wise score plots.

### 2.12. Pseudotime Trajectory Analysis

Epithelial cells were subsetted, and inferCNV-derived CNV scores were used to assign high, intermediate, and low-malignancy states, which were visualized on UMAP. An epithelial pseudotime trajectory was reconstructed using Monocle2 with DDRTree, and orig.ident was regressed out to mitigate sample-driven effects. Trajectories were visualized using pseudotime, CNV score, and malignancy state. Pseudotime and state assignments were subsequently transferred back to the Seurat object metadata for downstream analyses.

### 2.13. Spatial Transcriptomic Integration and Label Transfer

Spatial transcriptomic data were obtained from the NCBI BioProject PRJNA1124001, with tissue images and metadata retrieved from Zenodo (https://zenodo.org/records/13379726, accessed on 25 January 2026). Raw FASTQ files were processed using 10× Genomics Cell Ranger (Visium) to generate standard Visium outputs, and a SingleR-annotated scRNA-seq object was used as a reference for mapping. Eight PDAC Visium samples were loaded into Seurat, filtered to remove zero-count spots (nCount_Spatial > 0 and nFeature_Spatial > 0), normalized with SCTransform, and integrated using the SCT workflow (3000 features), followed by PCA/UMAP (dims = 1:30). Spot-level cell types were inferred by Seurat anchor-based label transfer (dims = 1:30) to obtain predicted.id, and the epithelial prediction score (Epithelial_score) was extracted for downstream analyses.

### 2.14. DKK1 Program Scoring and CellChat Communication Analysis

In scRNA-seq epithelial cells, a DKK1-associated module was defined by filtering lowly expressed genes (mean expression > 0.01), computing Pearson correlations with DKK1, and selecting the top 50 positively correlated genes (excluding DKK1). This gene set was projected onto the integrated spatial dataset, and spot-level DKK1 program scores (DKK1_prog) were computed using Seurat’s AddModuleScore on SCT-normalized data (nbin = 24; ctrl = 5) and visualized spatially. Cell–cell communication was inferred using CellChat (CellChatDB.human) with spot-level predicted.id used for cell-type labels (min.cells = 10); the results were summarized using global circle plots and pathway-level bubble plots.

### 2.15. Cell Culture

The human pancreatic cancer cell lines MIA PaCa-2 and PANC-1 were obtained from the American Type Culture Collection (ATCC; Manassas, VA, USA) and maintained in DMEM (Gibco, 11965092, Waltham, MA, USA) supplemented with 10% FBS (Gibco, 10099141) and 1% penicillin–streptomycin (Gibco, 15140122) at 37 °C in a humidified 5% CO_2_ incubator. Cells were routinely passaged at 80–90% confluence using 0.25% trypsin–EDTA (Gibco, 25200056) and reseeded at ~1 × 10^5^ cells per flask, with routine mycoplasma testing.

### 2.16. Lentiviral shRNA Transduction and Generation of Stable DKK1-Knockdown Cell Lines

Stable DKK1-knockdown cell lines were generated using a lentiviral shRNA approach. Two shRNAs targeting human DKK1 were custom-synthesized by RiboBio Co., Ltd. (Guangzhou, China). In brief, HEK293T cells were transfected with the lentiviral shRNA plasmids targeting DKK1, along with packaging plasmids using Lipofectamine™ 3000 (Invitrogen, Waltham, MA, USA) according to the manufacturer’s instructions. Viral supernatants were collected at 48 and 72 h post-transfection and passed through a 0.45 μm filter. Target pancreatic cancer cells were transduced with the viral supernatant, and the medium was replaced with fresh complete medium after transduction. Puromycin selection was initiated 48 h after transduction at 2 μg/mL and maintained for 2 weeks to obtain stable knockdown cell populations.

The shRNA sequence for targeting DKK1 were GTATGTGTGTGTTCTACAACTCGAGTTGTAGAACACACACATAC for shRNA1 and GGAAGTGTGATATGTTTAACTCGAGTTAAACATATCACACTT-CC for shRNA2.

### 2.17. Cell Counting Kit-8 (CCK-8) Assay

Cell viability/proliferation was measured using the CCK-8 (Dojindo, Cat. No. CK04, Tokyo, Japan) assay according to the manufacturer’s instructions. MIA PaCa-2 and PANC-1 cells were seeded in 96-well plates at 5 × 10^3^ cells per well in 100 μL complete medium and incubated at 37 °C with 5% CO_2_. At 24, 48, 72, and 96 h, 10 μL CCK-8 reagent was added to each well and incubated for 2 h, and absorbance at 450 nm was recorded using a microplate reader (BioTek Synergy H1, Winooski, VT, USA). Proliferation was expressed as the absorbance in treated wells relative to control wells.

### 2.18. Colony Formation

Colony formation assays were performed using MIA PaCa-2 and PANC-1 cells. Cells were seeded in 6-well plates (500 cells/well) and cultured for 10–14 days with medium changes every 3 days. Colonies (>50 cells) were fixed with 4% paraformaldehyde, stained with 0.1% crystal violet, and imaged (Leica DM3000, Wetzlar, Germany); subsequently, they were counted to calculate colony formation efficiency (colonies/seeded cells).

### 2.19. Transwell Migration and Invasion Assays

Transwell migration assays were performed using 8 μm pore inserts (Corning, 3422, Corning, NY, USA). Cells (1 × 10^5^ cells/mL) in serum-free medium were seeded into the upper chamber (200 μL), with 10% FBS-containing medium placed in the lower chamber as a chemoattractant. After 24 h, migrated cells were fixed (4% paraformaldehyde), stained with crystal violet (0.1%), and counted in five random fields per insert (Leica DM3000).

### 2.20. Wound-Healing Assay

Wound-healing assays were performed to assess cell migration. MIA PaCa-2 and PANC-1 cells were grown to near-confluence in 6-well plates, and a linear scratch was generated using a sterile 200 μL pipette tip, followed by replacement with serum-free medium (Gibco, 31603-029); images were acquired at 0 and 24 h (Leica DM3000). Wound closure was quantified using ImageJ (version 1.54) and calculated as:


$$Wound\;closure\;(\%)=({Gap}_{0}h-{Gap}_{24}h)/{Gap}_{0}h\times 100$$

### 2.21. Apoptosis Assay

Apoptosis was assessed using Annexin V–FITC/PI staining (BD Biosciences, 556547, San Jose, CA, USA) followed by flow cytometry. Treated MIA PaCa-2 and PANC-1 cells were harvested, washed, resuspended in binding buffer, and stained with Annexin V–FITC (5 μL) and PI (5 μL) for 15 min at room temperature in the dark. Samples were analyzed on a BD FACSCalibur and quantified in FlowJo v10, with apoptosis defined as Annexin V^+^/PI^−^ (early) plus Annexin V^+^/PI^+^ (late).

### 2.22. Western Blot

Total proteins were extracted from the normal cell, tumor cell, shDKK1-1, and shDKK1-2 groups using a total protein extraction kit (P0037, Beyotime, Shanghai, China) and quantified with a BCA protein assay kit (ZJ101, Yeasen Biotechnology, Shanghai, China). Equal amounts of protein (40 μg per sample) were separated using 10% SDS–PAGE and transferred onto PVDF membranes (0.45 μm; MilliporeSigma, Burlington, MA, USA). Subsequently, the membranes were blocked with 5% (*w*/*v*) non-fat milk in TBST (TBS containing 0.05% Tween-20) for 1 h at room temperature and incubated overnight at 4 °C with the following primary antibodies: rabbit anti-β-catenin (1:1000; Cell Signaling Technology, Danvers, MA, USA; 8480), rabbit anti-cleaved caspase-3 (1:1000; Cell Signaling Technology; 9664), and mouse anti-β-actin (1:20,000; Proteintech, Wuhan, China; 66009-1-Ig). After washing, membranes were incubated with an HRP-conjugated secondary antibody (1:10,000; Proteintech; SA00001-2) for 1 h at room temperature. Signals were developed using an ECL substrate (SQ201, Yeasen Biotechnology, Shanghai, China) and imaged on an Amersham™ ImageQuant™ 800 system (Cytiva, Marlborough, MA, USA). Band intensities were semi-quantified based on chemiluminescence signals.

### 2.23. Statistical Analysis

Statistical analyses were performed using SPSS (v26.0; IBM Corp., Armonk, NY, USA) and R (V4.3.1). Data are presented as mean ± standard deviation (SD) for normally distributed variables and as median (interquartile range, IQR) for non-normally distributed variables. Normality was assessed using the Shapiro–Wilk test. Comparisons between two independent groups were performed using either Student’s *t* test (normal distribution) or the Mann–Whitney U test (nonparametric). Comparisons among more than two groups were conducted using one-way analysis of variance (ANOVA) followed by Tukey’s post hoc test. Correlations were evaluated using Pearson’s or Spearman’s correlation, as appropriate.

## 3. Results

### 3.1. The Workflow of This Study

**Figure 1:**
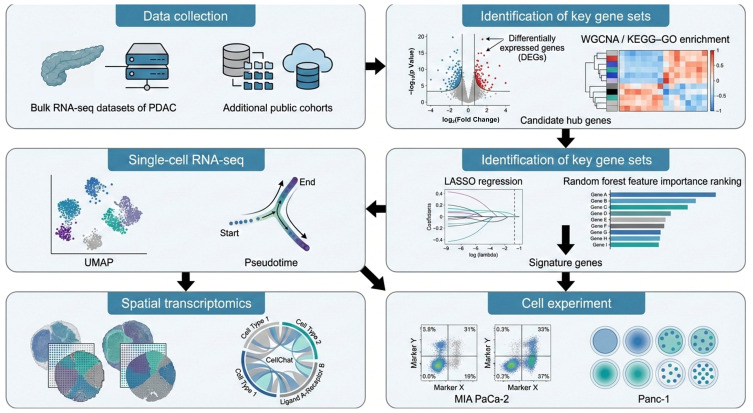
Workflow for the identification and validation of signature genes in PDAC.

In this study, we reanalyzed multiple publicly available cohorts to validate the predictive genes that we had identified. Candidate genes were first derived from differential expression analysis of the bulk RNA dataset GSE15471 and then evaluated in an independent integrated bulk cohort combining TCGA-PAAD with GSE62452. To resolve cellular heterogeneity and the tumor microenvironmental context, we further integrated two scRNA-seq datasets (GSE205013 and GSE155698) and spatial transcriptomic data (PRJNA1124001) to map the tissue distribution of key molecular signals. The overall workflow is summarized in Figure 1.

### 3.2. Transcriptomic Profiling Identified Extracellular Matrix (ECM) Remodeling and Oncogenic Pathways in PDAC

**Figure 2:**
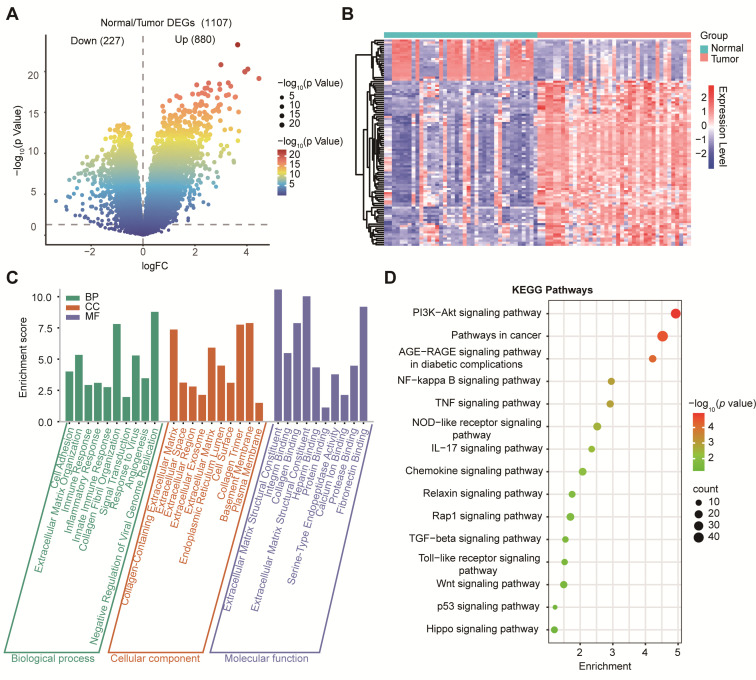
Differential gene expression and functional enrichment analysis in PDAC. (**A**) Volcano plot showing DEGs between tumor and normal tissues. (**B**) Heatmap of differential gene expression between PDAC tumors and normal pancreas. Heatmap showing the expression of the top 50 DEGs between tumor (n = 39) and normal (n = 39) samples from GSE15471. Columns represent individual samples, annotated by group (Normal vs. Tumor). Rows represent genes. Expression values are shown as row-wise z-scores of log2(TPM + 1) (color scale), where red indicates higher and blue indicates lower relative expression. Genes were selected based on limma using |log2FC| ≥ 1 and *p* < 0.05. Both genes and samples were hierarchically clustered using Pearson correlation. (**C**) GO enrichment analysis of DEGs categorized into BP, CC, and MF. (**D**) KEGG pathway enrichment analysis highlighting significantly enriched signaling pathways.

Our differential expression analysis of GSE15471 identified 1107 DEGs, including 880 upregulated and 227 downregulated genes (|log_2_FC| > 1, *p* < 0.05; Figure 2A). To visualize global transcriptional differences between PDAC tumors and normal pancreas samples, we generated a heatmap of the top 50 differentially expressed genes (DEGs) identified from GSE15471 (Figure 2B). Expression values were log2(TPM + 1) transformed and standardized row-wise (z-score) across samples to highlight relative up- and down-regulation patterns. Unsupervised hierarchical clustering revealed a clear separation between tumors and normal samples, with tumors showing a coordinated upregulation of tumor-associated programs compared to normal samples, which displayed distinct baseline expression profiles. GO biological process terms were enriched for ECM organization, immune regulation, and cell adhesion; cellular component terms were enriched for the extracellular region and collagen-containing matrix; and molecular function terms were enriched for integrin binding and cytokine activity (Figure 2C). KEGG analysis further implicated cancer-related pathways, including PI3K–Akt, NF-κB, IL-17, TGF-β, and Wnt signaling (Figure 2D), consistent with stromal remodeling and inflammatory/oncogenic activation in PDAC.

### 3.3. WGCNA Identified the Tumor-Associated Module and Yielded 263 Hub Genes in PDAC

WGCNA was performed to identify gene co-expression modules associated with PDAC.

**Figure 3:**
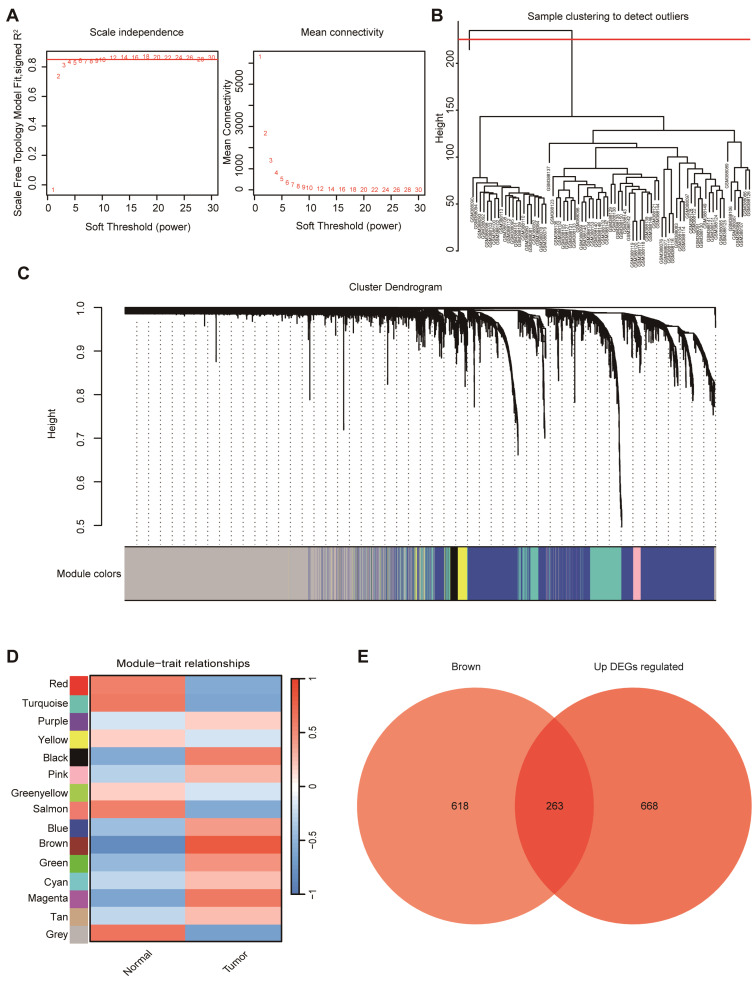
WGCNA of PDAC samples. (**A**) Determination of soft-thresholding power based on scale independence and mean connectivity to ensure a scale-free network topology. (**B**) Sample clustering dendrogram used to identify potential outliers. (**C**) Gene clustering dendrogram with module color assignments generated by WGCNA. (**D**) Module–trait relationship heatmap showing correlations between gene modules and clinical traits (normal vs. tumor). (**E**) Venn diagram displaying the overlap between in the brown module and up-regulated DEGs.

A soft-thresholding power of 6 was selected to satisfy the scale-free topology criterion (Figure 3A). Sample clustering identified one outlier (GSM388111), which was excluded, leaving 77 samples for downstream analysis (Figure 3B). A hierarchical clustering dendrogram of genes was generated based on topological overlap, with dynamic tree cutting delineating discrete co-expression modules (Figure 3C). The clear separation of major branches supports stable module assignments for subsequent module–trait association analyses. Among the fifteen co-expression modules identified, the brown module (MEbrown) showed the strongest association with tumor status (r = 0.81, *p* < 0.0001) genes and up-regulated DEGs (Figure 3D). To further refine candidates, genes in MEbrown were intersected with upregulated DEGs, yielding 263 overlapping genes (Figure 3E), which were carried forward as hub genes for subsequent prioritization.

### 3.4. Integration of LASSO and RF Identified Four Candidate Genes in PDAC

**Figure 4:**
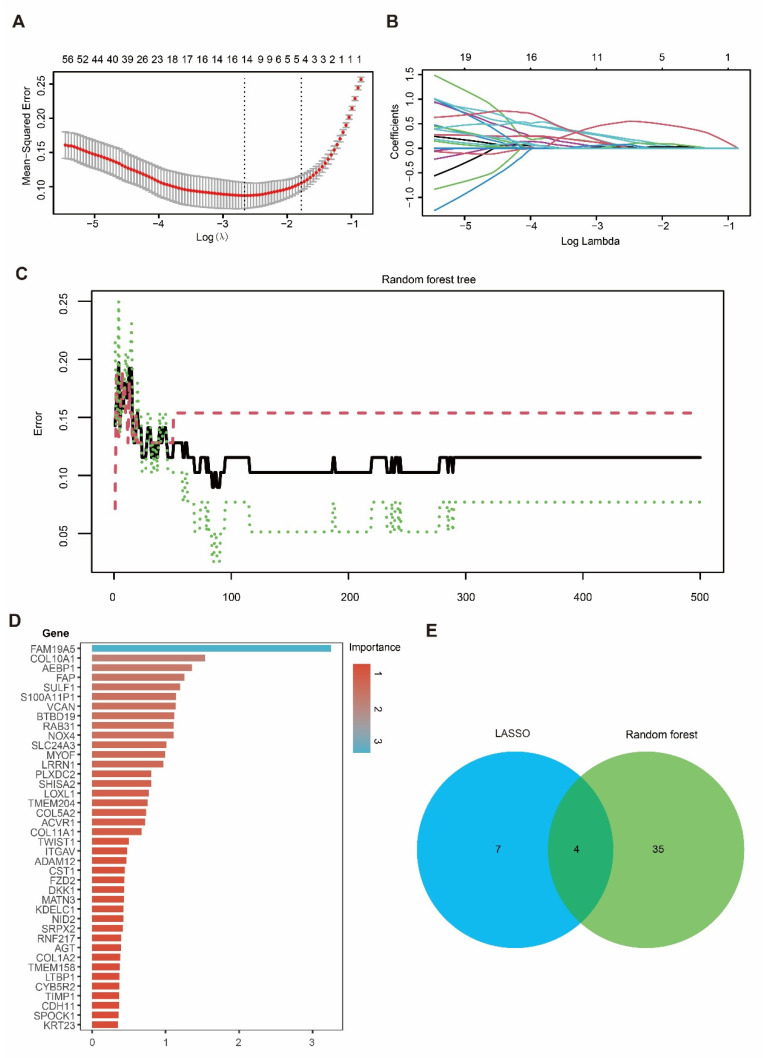
Identification of candidate hub genes using LASSO and RF algorithms. (**A**) Ten-fold cross-validation for tuning parameter (λ) selection in the LASSO regression model. (**B**) LASSO coefficient profiles of genes as a function of the regularization parameter λ. (**C**) Error rate curve of the Random Forest model showing classification accuracy across different tree numbers. (**D**) Importance ranking of candidate genes identified by the Random Forest model. (**E**) Venn diagram showing the overlap of candidate hub genes selected by LASSO and Random Forest, with four genes identified by both methods.

To prioritize the candidate genes, we applied LASSO regression and RF to the hub gene set. In the LASSO model, the optimal penalty parameter (λ) was selected using five-fold cross-validation to minimize the mean squared error (Figure 4A), yielding 11 genes with non-zero coefficients at the selected λ (Figure 4B). In parallel, an RF classifier (500 trees) showed stabilized error rates with increasing tree numbers (Figure 4C), and variable-importance analysis highlighted genes contributing to tumor–normal discrimination (Figure 4D). By intersecting the LASSO-selected genes with the top RF features, we identified four overlapping candidate genes—*DKK1*, *COL10A1*, *SULF1*, and *SLC24A3* (Figure 4E)—which were carried forward for downstream validation and functional analyses.

### 3.5. The Four Candidate Genes Are Consistently Upregulated in PDAC Tumors

**Figure 5:**
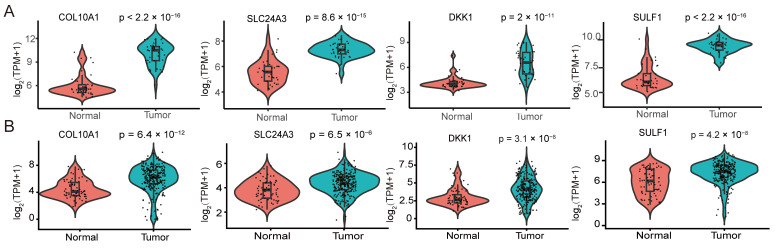
Differential expression of hub genes and prognostic performance of the four-gene risk signature in pancreatic ductal adenocarcinoma. (**A**) Violin plots showing the expression differences of the four hub genes COL10A1, SLC24A3, DKK1 and SULF1 between normal pancreatic tissues and PDAC tissues in the GSE15471 dataset. (**B**) Violin plots depicting the expression patterns of COL10A1, SLC24A3, DKK1 and SULF1 in normal versus tumor tissues in the TCGA-PAAD cohort and in the integrated GSE62452 validation cohort. Expression values are presented as log2-transformed TPM with a pseudocount of 1 [Expression = log2(TPM + 1)]. Each dot represents one sample; the embedded box indicates the median and interquartile range. *p* values were calculated using the Wilcoxon rank-sum test (two-sided).

We then compared the expression of the four candidate genes between PDAC tumors and non-tumor tissues. In the GSE15471 discovery cohort, COL10A1, SLC24A3, DKK1, and SULF1 were all significantly upregulated in tumor samples (Figure 5A). In the independent validation cohort integrating TCGA-PAAD and GSE62452, all four genes again showed higher expression in tumors, recapitulating the pattern observed in GSE15471 (Figure 5B). Collectively, these results demonstrate the reproducible tumor-associated upregulation of COL10A1, SLC24A3, DKK1, and SULF1 across datasets and platforms.

### 3.6. Single-Cell Analysis Links Epithelial-Derived DKK1 to Malignant Progression and Microenvironment Remodeling

**Figure 6:**
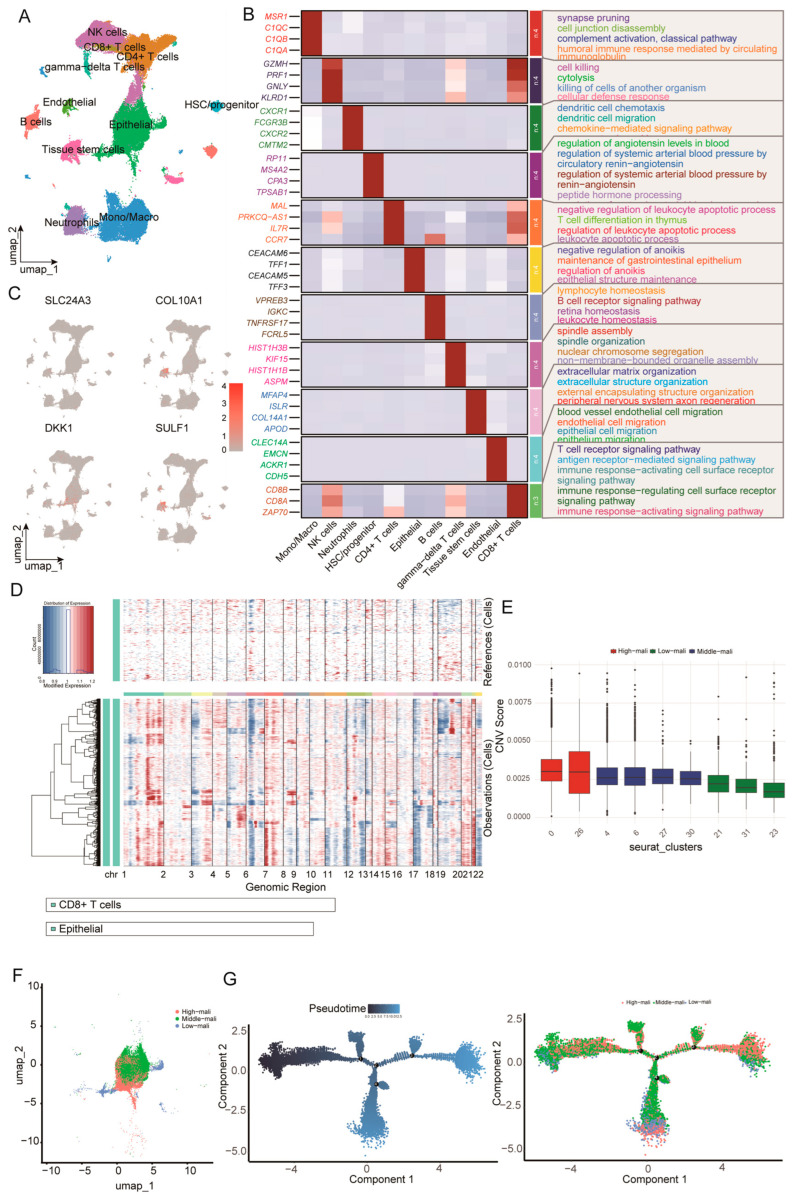
Single-cell reference atlas, malignant epithelial cell identification, and trajectory analysis. (**A**) UMAP of the integrated scRNA-seq dataset showing major tumor microenvironment cell populations. (**B**) Heatmap of representative marker genes across major lineages with enriched GO biological processes annotated. (**C**) UMAP feature plots showing normalized expression of SLC24A3, COL10A1, DKK1, and SULF1. (**D**) Large-scale CNV profiles inferred by inferCNV, with reference cells shown at the top and epithelial cells at the bottom across chromosomes 1–22. (**E**) Boxplots of CNV scores across epithelial Seurat clusters used to define high, middle, and low-malignant states. (**F**) UMAP of epithelial cells colored by malignant state. (**G**) Monocle pseudotime trajectory of epithelial cells colored by pseudotime and malignant state.

We integrated 27 primary PDAC scRNA-seq samples (GSE205013 and GSE155698), yielding 154,590 cells, and retained 95,218 high-quality cells after quality control. Dimensionality reduction (PCA/UMAP) resolved the major cellular compartments of the PDAC tumor microenvironment, including epithelial, endothelial, stem/progenitor, and immune populations (Figure 6A). Cluster-specific markers supported these annotations and revealed lineage-associated transcriptional programs (Figure 6B).

We next mapped the four candidate genes onto the single-cell atlas (Figure 6C). Among the four candidates, DKK1 exhibited the most pronounced cell-type specificity, with predominant expression in epithelial cells and minimal expression across immune populations, indicating that the tumor-associated epithelium is a major cellular source. To distinguish malignant from non-malignant epithelial states, we inferred large-scale CNV profiles using inferCNV with CD8^+^ T cells (and non-epithelial populations) as reference cells (Figure 6D). Epithelial cells were stratified into low, intermediate, and high-malignancy states based on CNV burden, which formed a continuum with partially separated distributions in UMAP space (Figure 6F). Monocle2 pseudotime analysis, rooted in the low-malignancy state, further supported a progressive trajectory from early to late states, with high-malignancy cells enriched at late pseudotime (Figure 6G). Taken as a whole, our results demonstrate the continuum of epithelial malignant progression characterized by increasing CNV burden and coordinated transcriptional reprogramming, and they place epithelial-derived DKK1 within this framework.

Consistent with our single-cell observations, bulk transcriptomic analyses linked DKK1 to tumor-intrinsic and microenvironmental programs. Single-gene GSEA in GSE15471 showed that higher DKK1 expression was associated with enrichment of the cell cycle, ECM–receptor interaction, and focal adhesion pathways (Figure S1A). Immune deconvolution further indicated positive correlations between DKK1 and myeloid populations (macrophage subsets and neutrophils), alongside negative correlations with cytotoxic lymphocytes, particularly CD8^+^ T cells and activated NK cells (Figure S1C). Collectively, these findings suggest that epithelial-derived DKK1 co-occurs with pro-tumor transcriptional programs and an immune contexture dominated by myeloid infiltration and reduced cytotoxic activity.

### 3.7. Spatial Heterogeneity of the DKK1-Associated Program and Its Relationship to Cell–Cell Communication

**Figure 7:**
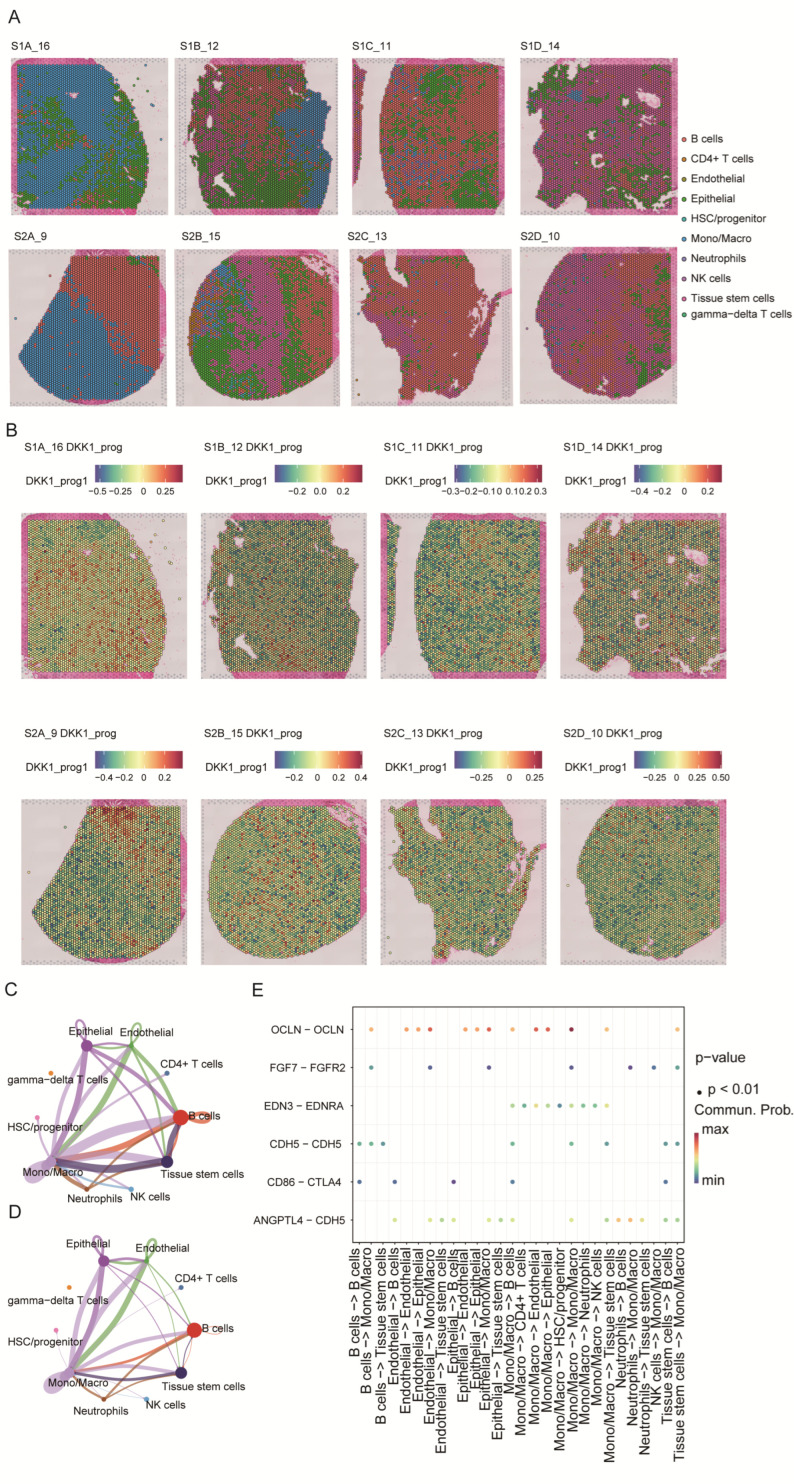
Spatial distribution of the DKK1-associated program and its inferred cell–cell communication. (**A**) Spatial cell-type mapping of eight primary PDAC Visium sections by label transfer from the single-cell reference atlas. (**B**) Spatial distribution of the DKK1-associated module score (DKK1_prog) across sections. (**C**,**D**) Global cell–cell communication networks inferred by CellChat, with node size indicating overall signaling strength and edges representing interaction probability/strength between cell types. (**E**) Dot plot of selected ligand–receptor pairs related to epithelial–endothelial barrier regulation and immune modulation across sender–receiver cell-type pairs; dot color denotes communication probability and dot size indicates statistical significance.

Using the integrated scRNA-seq reference and Seurat label transfer, we mapped major cell types onto eight primary PDAC Visium sections. Epithelial-enriched spots localized predominantly to tumor parenchyma, whereas monocytes/macrophages, lymphocytes, and neutrophils were preferentially distributed in stromal and tumor-margin regions; endothelial cells and stem/progenitor populations showed more focal patterns (Figure 7A).

Because spot-level DKK1 expression was inconsistently detected across sections, we quantified a spot-level DKK1-associated program score (DKK1_prog) using the scRNA-seq-derived DKK1 gene module (Figure 7B). DKK1_prog displayed pronounced intratumoral heterogeneity and was enriched in epithelial-dominant regions relative to immune/stroma-dominant areas, supporting spatial coupling between the DKK1 program and the malignant epithelial niche. We next applied CellChat to the spatially annotated data to infer communication networks. Multiple cell types—including epithelial cells, endothelial cells, monocytes/macrophages, B cells, and stem/progenitor populations—emerged as major signaling hubs, with prominent interactions involving the epithelial–endothelial and endothelial–immune compartments (Figure 7C,D). At the ligand–receptor level, epithelial–endothelial interactions (e.g., OCLN–OCLN, CDH5–CDH5, and ANGPTL4–CDH5) and CD86–CTLA4 signaling between antigen-presenting/B cells and T cells were highlighted (Figure 7E). Collectively, these results indicate that DKK1_prog is enriched in epithelial-dominant regions and co-occurs with key epithelial–endothelial–immune communication patterns in PDAC.

### 3.8. DKK1 Knockdown Impairs Malignant Phenotypes and Promotes Apoptosis in Pancreatic Cancer Cells

**Figure 8:**
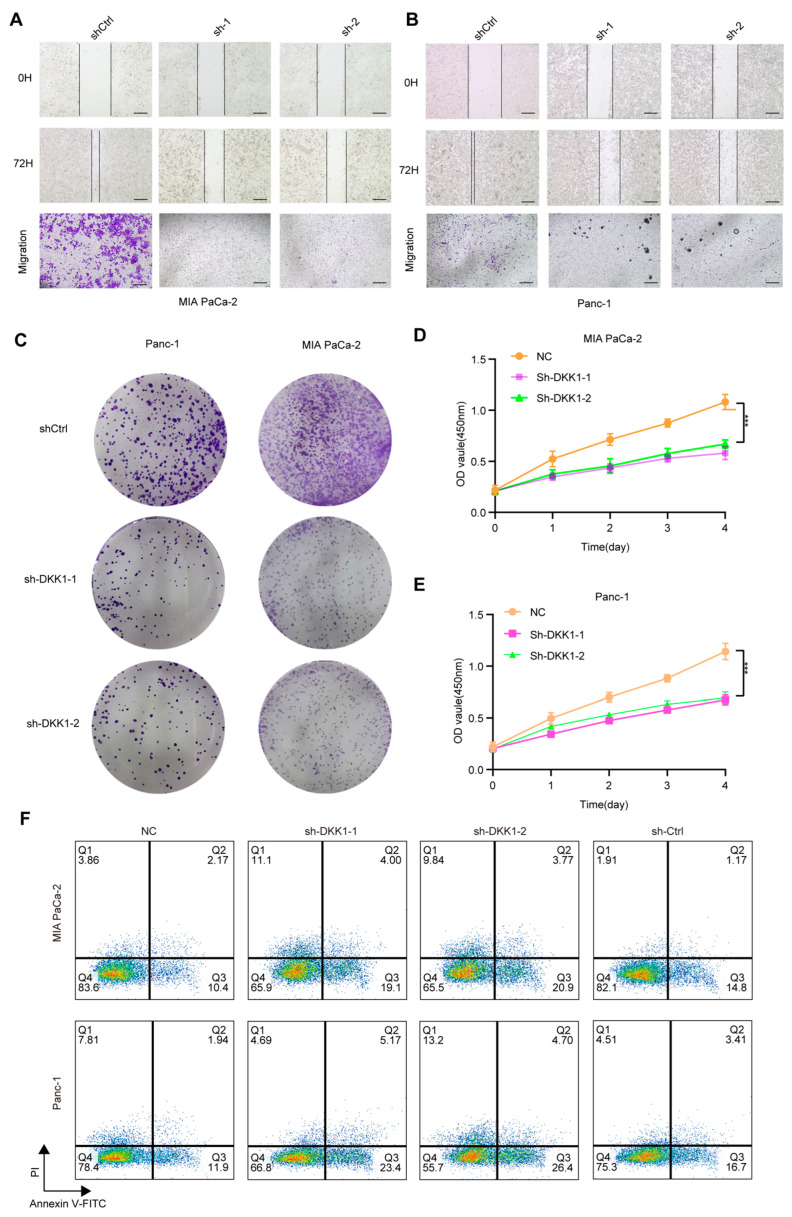
Functional effects of DKK1 knockdown in pancreatic cancer cell lines. (**A**,**B**) Wound-healing and Transwell migration assays showing reduced migratory ability of MIA PaCa-2 (**A**) and Panc-1 (**B**) cells after DKK1 knockdown compared with control. (**C**) Colony formation assays in Panc-1 and MIA PaCa-2 cells demonstrating decreased clonogenic capacity upon DKK1 silencing, images show whole wells of a 6-well plate (well diameter ≈ 35 mm). (**D**,**E**) CCK-8 proliferation assays showing significantly inhibited growth in MIA PaCa-2 (**D**) and Panc-1 (**E**) cells following DKK1 knockdown. (**F**) Flow cytometry analysis of apoptosis in MIA PaCa-2 and Panc-1 cells, indicating increased apoptotic rates in DKK1-silenced groups relative to controls. *** *p* < 0.001. Scale bars: 500 µm (**A**,**B**).

To functionally validate DKK1 in pancreatic cancer cells, we silenced DKK1 in MIA PaCa-2 and PANC-1 cells and assessed key malignant phenotypes. Wound-healing assays showed reduced migratory capacity following DKK1 knockdown, as indicated by delayed scratch closure compared with controls (Figure 8A,B). Transwell assays consistently showed fewer migrated cells upon DKK1 silencing (Figure 8A,B). Beyond migration, DKK1 knockdown decreased clonogenic growth in colony formation assays (Figure 8C) and suppressed cell proliferation over four days in CCK-8 assays (Figure 8D,E). We next performed flow-cytometric Annexin V/PI staining: Cells were first gated on the lymphocyte population based on FSC-A versus SSC-A and then filtered to single cells using FSC-A versus FSC-H to exclude doublets. Apoptosis was subsequently quantified within singlets using Annexin V–FITC/PI staining with quadrant analysis to define viable (Annexin V^−^/PI^−^), early apoptotic (Annexin V^+^/PI^−^), late apoptotic/secondary necrotic (Annexin V^+^/PI^+^), and necrotic (Annexin V^−^/PI^+^) cells (Figure S2A). Flow-cytometric Annexin V–FITC/PI staining showed that DKK1 silencing increased apoptosis in both PDAC cell lines. In MIAPaCa-2 cells, the early apoptotic fraction (Annexin V^+^/PI^−^) increased from 10.4% (NC) and 14.8% (sh-Ctrl) to 19.1% (sh-DKK1-1) and 20.9% (sh-DKK1-2), while the late apoptotic fraction (Annexin V^+^/PI^+^) rose from 2.17% (NC) and 1.17% (sh-Ctrl) to 4.00% (sh-DKK1-1) and 3.77% (sh-DKK1-2); the necrotic fraction (Annexin V^−^/PI^+^) was 3.86% (NC), 1.91% (sh-Ctrl), 11.1% (sh-DKK1-1), and 9.84% (sh-DKK1-2). Similarly, in Panc-1 cells, early apoptosis increased from 11.9% (NC) and 16.7% (sh-Ctrl) to 23.4% (sh-DKK1-1) and 26.4% (sh-DKK1-2), and late apoptosis increased from 1.94% (NC) and 3.41% (sh-Ctrl) to 5.17% (sh-DKK1-1) and 4.70% (sh-DKK1-2); the necrotic fraction was 7.81% (NC), 4.51% (sh-Ctrl), 4.69% (sh-DKK1-1), and 13.2% (sh-DKK1-2) (Figure 8F). Flow cytometric Annexin V/PI staining further revealed increased apoptosis after DKK1 silencing, with elevations in both early and late apoptotic fractions (Figure 8F). We also observed a significant increase in cleaved caspase-3 in pancreatic cancer cells with DKK1 knockout (Figure S2B). In contrast, Lan Shao et al. (2024) reported that DKK1 expression in PDAC is highly dependent on DKK1-SE activity [17], observing a marked decrease in DKK1 expression following DKK1-SE knockout and noted enrichment of Wnt-related pathways post-knockout, which are closely linked to DKK1 activity. Therefore, we treated pancreatic cancer cells with shRNA and analyzed Wnt pathway expression in vivo after DKK1 knockout by detecting β-catenin protein expression (Figure S2B). Collectively, these results indicate that DKK1 supports the migration and growth of pancreatic cancer cells and restrains apoptosis.

## 4. Discussion

PDAC remains among the most lethal malignancies, with a 5-year survival rate below 10% despite advances in surgical techniques and systemic therapies [20,21,22]. Standard-of-care modalities—including surgery, chemotherapy, radiotherapy, and emerging immunotherapeutic approaches—have yielded only incremental improvements [23,24]. In contrast to several other solid tumors, PDAC responds poorly to immune checkpoint blockade, largely owing to its dense desmoplastic stroma, low tumor mutational burden, and profoundly immunosuppressive microenvironment [25]. Together, these features highlight an unmet need to define actionable molecular drivers that mechanistically connect stromal dysregulation to adaptive immune suppression.

In this study, we applied an integrative framework combining bulk transcriptomic discovery, network modeling, and machine-learning prioritization with single-cell/spatial localization and functional perturbation to identify regulators associated with malignant progression and microenvironmental remodeling in PDAC. By integrating WGCNA with machine-learning prioritization, we distilled four reproducible hub genes (DKK1, COL10A1, SULF1, and SLC24A3) and validated them across independent datasets (Figure 3, Figure 4 and Figure 5). Among these prioritized candidates, DKK1 emerged as an epithelial-associated regulator: across bulk cohorts, it was consistently upregulated and embedded within tumor-associated programs enriched for ECM and adhesion-related pathways, while immune deconvolution linked higher DKK1 to increased myeloid infiltration and reduced cytotoxic lymphocyte signatures (Figure 2C,D and Figure S1A,C). Consistent with prior evidence implicating DKK1 in PDAC progression and microenvironmental remodeling [17], our study extends these observations by resolving its cellular origin, malignant-state positioning, and spatial communication using single-cell and spatial transcriptomics. At single-cell resolution, DKK1 showed pronounced epithelial specificity and aligned with an inferCNV and pseudotime-defined continuum of epithelial malignant progression (Figure 6C–G). Building on recent spatial profiling efforts that highlight architecture-constrained, fibrotic and immunosuppressive PDAC ecotypes, our spatial analyses extend these concepts by using a DKK1-associated program score to overcome sparse single-gene detection and to map marked intratumoral heterogeneity to epithelial-dominant regions [26] (Figure 7A,B). Our CellChat inference consistently highlighted epithelial–endothelial and endothelial–immune communication patterns co-occurring with high program activity, providing a tissue-contextual hypothesis for how tumor-intrinsic programs interface with the microenvironment (Figure 7C–E). Mechanistically, our observations are concordant with emerging evidence that aberrant DKK1 activation can be sustained by upstream regulatory elements (e.g., an AP-1–engaged DKK1 super-enhancer in PDAC) and that DKK1 can function as a microenvironmental modulator beyond canonical Wnt antagonism, including regulation of innate immune compartments and NK-cell activity [27,28]. Functionally, DKK1 knockdown impaired pancreatic cancer cell migration, proliferation, and clonogenic growth while increasing apoptosis, supporting a tumor-promoting role (Figure 8). These findings are consistent with prior reports implicating DKK1 in immune evasion and therapy resistance in gastrointestinal malignancies [16,29,30], but differ by providing a multi-scale, PDAC-specific framework that links an epithelial-derived DKK1 program to malignant state transitions, spatial tissue architecture, and inferred communication networks—features that may inform biomarker stratification and therapeutic evaluation of the DKK1–CKAP4 axis [31].

There are several limitations to our study which warrant consideration. First, our bulk analyses were retrospective and may have been influenced by cohort heterogeneity, tumor purity, and variable stromal content. Although cross-cohort validation improves robustness, prospective cohorts with harmonized clinical annotation will be required to establish clinical utility. Second, spatial transcriptomic profiling was performed at spot resolution, such that DKK1_prog and inferred communication networks may reflect mixed-cell signals. Moreover, CellChat provides probabilistic ligand–receptor inference and does not establish functional signaling. In future studies, orthogonal validation using multiplex imaging, spatial proteomics, or perturbation-based co-culture systems will be necessary. Third, our functional experiments were conducted in established PDAC cell lines and therefore do not recapitulate stromal and immune components that may condition DKK1-associated microenvironmental effects. Accordingly, microenvironment-aware platforms and in vivo models-including subcutaneous and/or orthotopic xenograft studies using stable DKK1-perturbed (e.g., knockdown/knockout) PDAC cells-are needed to physiologically validate the causal role of DKK1 in malignant progression and to test whether DKK1 inhibition remodels ECM states, vascular interactions, and immune infiltration. Fourth, while we observed apoptosis-related phenotypes upon DKK1 perturbation, we did not directly interrogate upstream apoptotic signaling to distinguish intrinsic versus extrinsic contributions (e.g., initiator caspases such as caspase-8/9/10). Future studies should incorporate initiator caspase cleavage/activity assays and pathway-focused perturbations to better define the mechanism of DKK1-regulated apoptosis in PDAC cells. Finally, given that DKK1 is a secreted factor, it should be systematically evaluated in the circulation as a non-invasive biomarker for patient stratification and treatment monitoring in well-annotated cohorts.

## 5. Conclusions

In conclusion, by integrating cross-cohort bulk transcriptomics with single-cell and spatial analyses and orthogonal functional validation, our study delineates an epithelial-derived DKK1_prog score that is tightly coupled with malignant-state transitions and microenvironmental remodeling in PDAC. This multi-scale framework links tumor-intrinsic transcriptional programs to tissue architecture and inferred intercellular communication, providing a mechanistically informed context for understanding immune exclusion in PDAC. Clinically, the reproducible upregulation and spatial heterogeneity of DKK1 support its utility for patient stratification and biomarker development, while our perturbation data underscore its functional relevance in tumor cell aggressiveness. Together, these findings highlight DKK1’s potential as a candidate for therapeutic exploration, including rational combinations with stroma-modulating strategies and immunotherapy to reprogram the suppressive PDAC ecosystem.

## Data Availability

The data that support the findings of this study are available from the corresponding author upon reasonable request.

## Supplementary Materials

The following supporting information can be downloaded at: https://www.mdpi.com/article/10.3390/cimb48020182/s1.
