# Supplementary material for: Multi-Omic and Spatial Profiling Identifies an Epithelial DKK1 Associated with Microenvironmental Remodeling in Pancreatic Ductal Adenocarcinoma
Source: Curr Issues Mol Biol. 2026 Feb 5;48(2):182. doi: 10.3390/cimb48020182 (PMC12939099; doi:10.3390/cimb48020182)
Supplement: Supplementary file 1 [file cimb-48-00182-s001.zip › cimb-4083088-supplementary.pdf]

SUPPORTING FILES

Multi-omic and spatial profiling identifies an epithelial DKK1 associated with microenvironmental remodeling in pancreatic ductal adenocarcinoma

Jiajia Xu[1, 2, a], Kaiqiang Qian[3, a], Yanyu Ding[1, 2], Jianghao Cheng[1, 2], Xu Zhang[1, 2], Yong Huang[1, 2, *]and Bo Liu[1, 2, *]

[1] Department of Immunology, School of Basic Medical Sciences, Center for Big Data and Population Health of IHM, Anhui Medical University, Hefei, Anhui, 230032, China.

[2] Hefei Comprehensive National Science Center, Institute of Health and Medicine, Hefei 230093, China.

[3] School of Life Sciences, National Engineering Laboratory of Crop Stress Resistance Breeding, Anhui Agricultural University, Hefei, Anhui, 230036, China.

[a] These authors contributed equally to this work

*Correspondence:

Bo Liu

Email: liubo@ihm.ac.cn

Yong Huang

Email: jonghuang@ihm.ac.cn

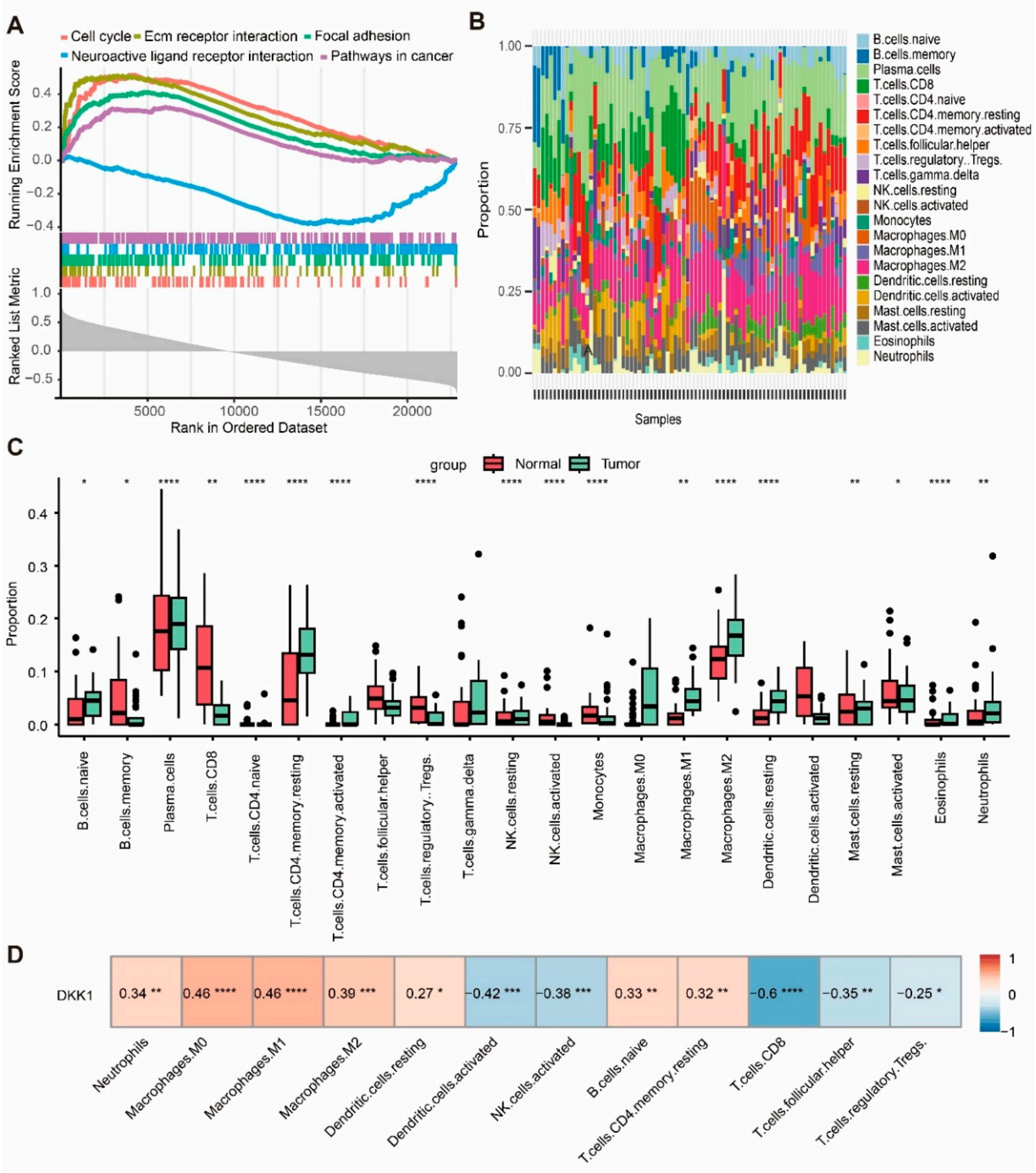

Figure S1. Functional enrichment and immune infiltration analysis of DKK1 in PDAC. (A) GSEA showing significant enrichment of pathways including cell cycle, ECM–receptor interaction, focal adhesion, neuroactive ligand-receptor interaction, and pathways in cancer. **(B)** Relative proportions of 22 tumor-infiltrating immune cell (TIIC) subtypes estimated by CIBERSORT in normal and tumor tissues. **(C)** Comparison of immune cell infiltration between normal and tumor samples, with significant increases in multiple immune subsets in tumors. **(D)** Correlation heatmap illustrating the association between DKK1 expression and immune cell infiltration levels. Memory resting T cells were positively correlated with DKK1 expression, whereas activated dendritic cells, activated NK cells, CD8^{+} T cells, follicular helper T cells (Tfh), and

regulatory T cells (Tregs) showed strong negative correlations with DKK1.

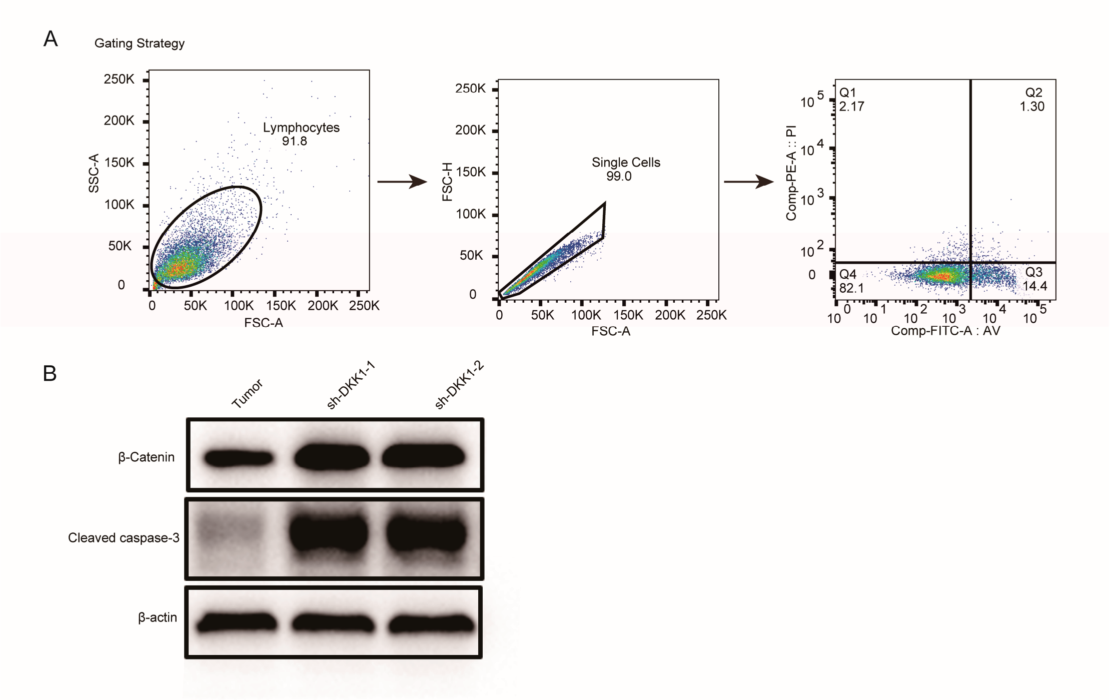

Figure S2. DKK1 knockdown promotes apoptosis and modulates Wnt signaling. (A) Representative flow-cytometry gating strategy. **(B)** Western blot analysis of β-catenin and cleaved caspase-3 in tumor cells transduced with control (Tumor) or DKK1 shRNAs (sh-DKK1-1 and sh-DKK1-2). β-actin was used as a control.
